# HIV-1 CRF07_BC Infections, Injecting Drug Users, Taiwan

**DOI:** 10.3201/eid1204.050762

**Published:** 2006-04

**Authors:** Yi-Ming Arthur Chen, Yu-Ching Lan, Shu-Fen Lai, Jyh-Yuan Yang, Su-Fen Tsai, Steve Hsu-Sung Kuo

**Affiliations:** * National Yang-Ming University, Taipei, Taiwan, Republic of China;; † Center for Disease Control, Taipei, Taiwan, Republic of China

**Keywords:** HIV-1, CRF07_BC, injecting drug user, harm reduction, Taiwan, letter

**To the Editor:** To date, Taiwan's human immunodeficiency virus type 1 (HIV-1) epidemic has primarily spread via sexual contact. The subtype B and circulating recombinant form (CRF) 01_AE account for >95% of all infections (*1*). However, since 2003 Taiwan has experienced a major outbreak of CRF07_BC among injecting drug users (IDUs).

The first wave of HIV-1 infections in Taiwan can be traced to the early 1980s, when a group of hemophilia patients received imported HIV-1-contaminated antihemophilia medications. By the time these medications had been replaced by heat-treated factor VIII concentrates, at least 53 patients had contracted HIV-1 infections (*2*). According to Taiwan's Center for Disease Control (CDC), HIV infections have been diagnosed in 9,229 persons (including 523 foreigners) as of July 31, 2005 (*3*). The number of persons living with HIV-1/AIDS has increased rapidly in the past few years, with a 77% increase in 2004, compared to 11% in 2003 (Table). According to the results of a risk factor analysis of people living with HIV-1/AIDS reported to the Taiwan CDC, the proportion of IDUs increased from 1.7% (13/773) in 2002 to 8.1% (70/861) in 2003 to 30.3% (462/1,521) in 2004 (Table). The Taiwan CDC received reports of 1,241 IDUs diagnosed with HIV-1 infections from January 1 to July 31, 2005; these account for >75% of all reported HIV-1 infections in 2005 (*3*). The evidence points to an explosive epidemic of HIV-1 infections among IDUs in Taiwan since 2003, with no indication of a slowdown.

**Table:** Annual number of HIV/AIDS cases reported to Taiwan Center for Disease Control, classified by risk group

Risk factors	No. case by year										
	1994	1995	1996	1997	1998	1999	2000	2001	2002	2003	2004
HIV+	174	228	277	350	401	479	535	654	773	859	1513
AIDS	63	98	159	135	152	179	179	161	178	227	254
Heterosexuals	85	109	134	154	189	214	235	275	310	251	244
Homosexuals	46	70	96	137	141	178	223	287	335	341	339
Bisexuals	27	41	37	47	59	70	61	64	79	51	54
Hemophiliacs	1	1	2	1	0	0	0	0	0	0	0
Injection drug users	6	6	6	5	3	1	7	4	13	70	462
Blood recipients	1	1	0	1	1	0	3	0	0	0	2
Vertical transmission	0	0	0	0	0	6	1	0	0	1	2
Unknown	8	0	2	5	8	10	5	24	36	145	410
Total	174	228	277	350	401	479	534	654	773	859	1513

Taiwan has ≈60,000 IDUs (*1*). According to the Republic of China Ministry of Justice, the number of incarcerated drug offenders increased from 5,988 in 2003 to 9,303 in 2004; the rate of HIV-1 seropositive inmates increased from 13.3/100,000 in 2002 to 56.8/100,000 in 2004 (Y-M. Wu, Ministry of Justice, pers. comm.). Since all inmates are routinely tested for HIV-1 in detention centers, and all infected inmates are separated from HIV-1-seronegative inmates, the potential of HIV-1 transmission in prisons is remote. We therefore suggest that the Taiwanese IDU population and its HIV-1 seropositive rate have both increased rapidly in the past few years.

To identify the primary HIV-1 strains in the current epidemic, we collected blood specimens from HIV-1-infected inmates in 3 detention centers (1 each located in the northern, central and southern regions of Taiwan). HIV-1 subtypes were determined by polymerase chain reaction, DNA sequencing, and phylogenetic analyses of *pol* or *env* genes. Our results indicate that 145 (96%) of 151 IDUs were infected with CRF07_BC and 6 (4%) were infected with subtype B; 97% of the CRF07_BC cases were diagnosed in 2003 or 2004. According to our phylogenetic analysis of the *env* gene, the Taiwanese CRF07_BC strains clustered with CRF07_BC strains drawn from IDUs in China (Figure).

**Figure:**
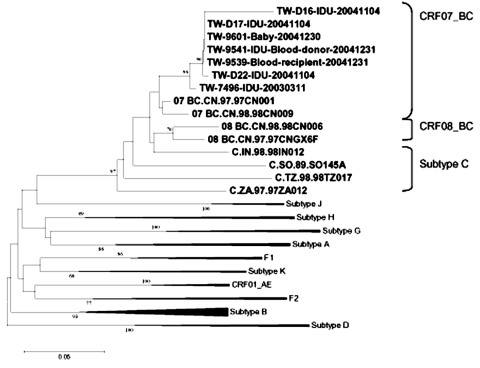
Phylogenetic analyses of 7 HIV-1 isolates identified in Taiwan. TW-7496, TW-D16, TW-D17, and TW-D22 were collected from detention center inmates; TW-9541 and TW-9539 were collected from a blood donor and 1 of his donation recipients. This neighbor-joining tree was created from 100 bootstrap samples of aligned *env* sequences corresponding to the 7077–7340 nucleotide residues of HIV-1-HXB2 from different isolates. Bootstrap values are shown on branch nodes. Reference isolates from the GenBank HIV database are indicated by subtype.

CRF07_BC is a recombinant of the B´ and C subtypes. Several studies have suggested that CRF07_BC originated in China's Yunnan Province, with subtype B´ from Thailand mixing with subtype C from India before moving northwestward to Xinjiang Province along a major Chinese heroin trafficking route (*4**–**6*). To our knowledge, this is the first report of a large group of IDUs in northeastern Asia having CRF07_BC infections. It may have followed another drug trafficking route from Yunnan Province to southeast China, moving through Guangxi Province and Hong Kong to Taiwan (*7**–**9*). In a bid to combat skyrocketing HIV/AIDS infection rates among IDUs, the Taiwan CDC has proposed a 5-year harm reduction program to the Republic of China Executive Yuan.
